# Supplementary figures and images for: Target Mechanisms of the Cyanotoxin Cylindrospermopsin in Immortalized Human Airway Epithelial Cells
Source: Toxins (Basel). 2022 Nov 11;14(11):785. doi: 10.3390/toxins14110785 (PMC9698144; doi:10.3390/toxins14110785)

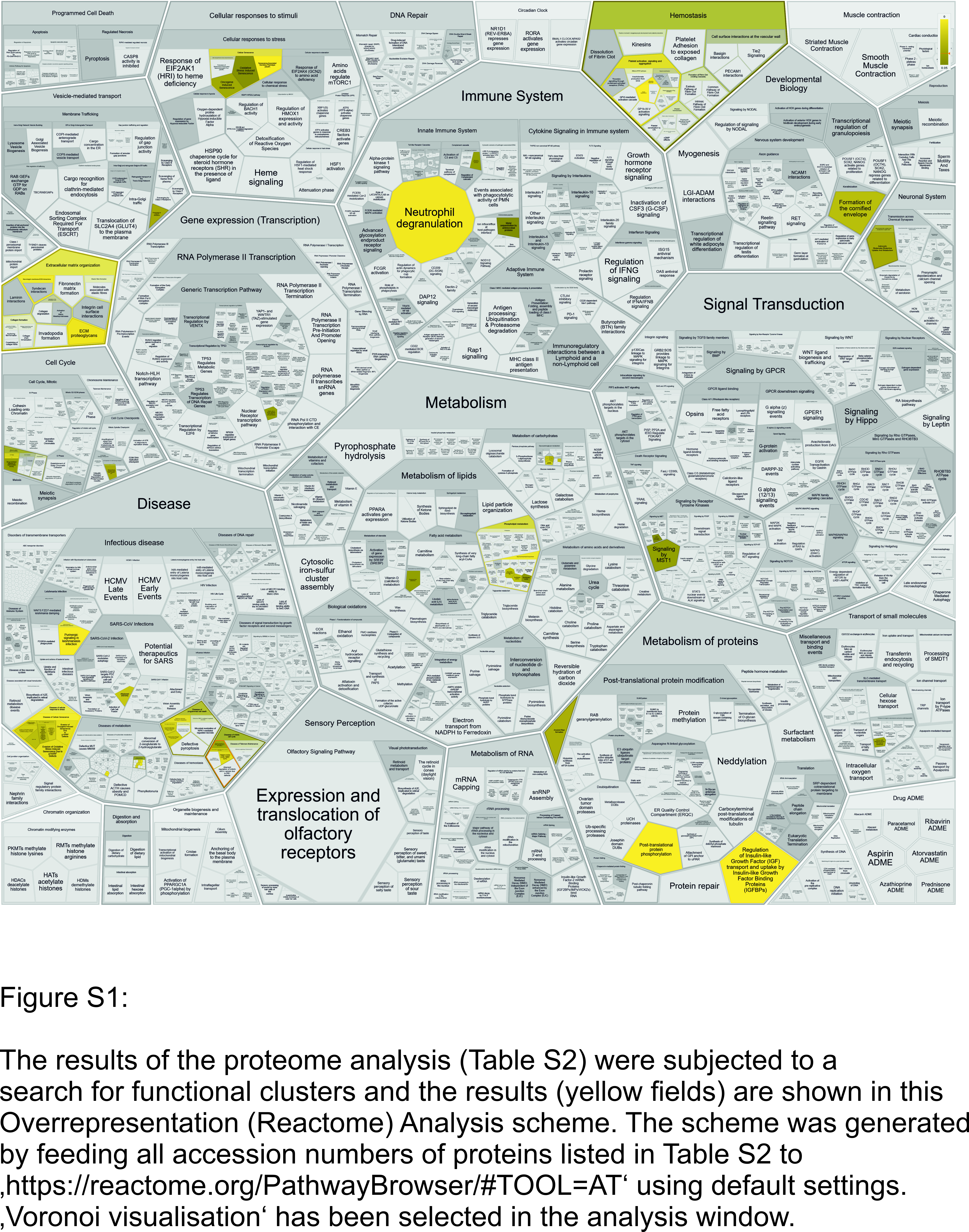

Supplement: Supplementary file 1 [file toxins-14-00785-s001.zip › Figure S1.tif]

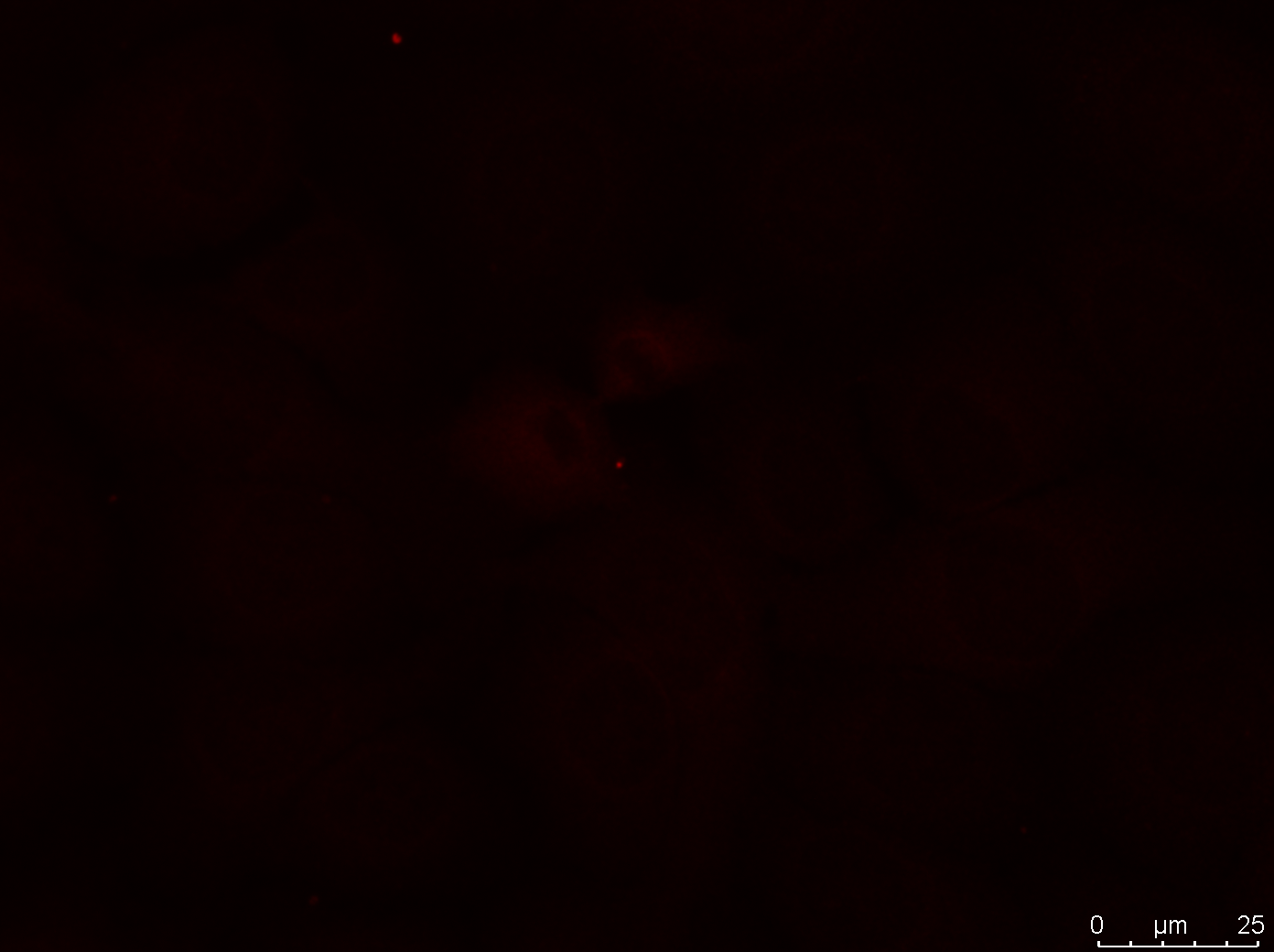

Supplement: Supplementary file 1 [file toxins-14-00785-s001.zip › to Fig 3_CEP55_Fluorescence_16HBE14o- Cells.tif]

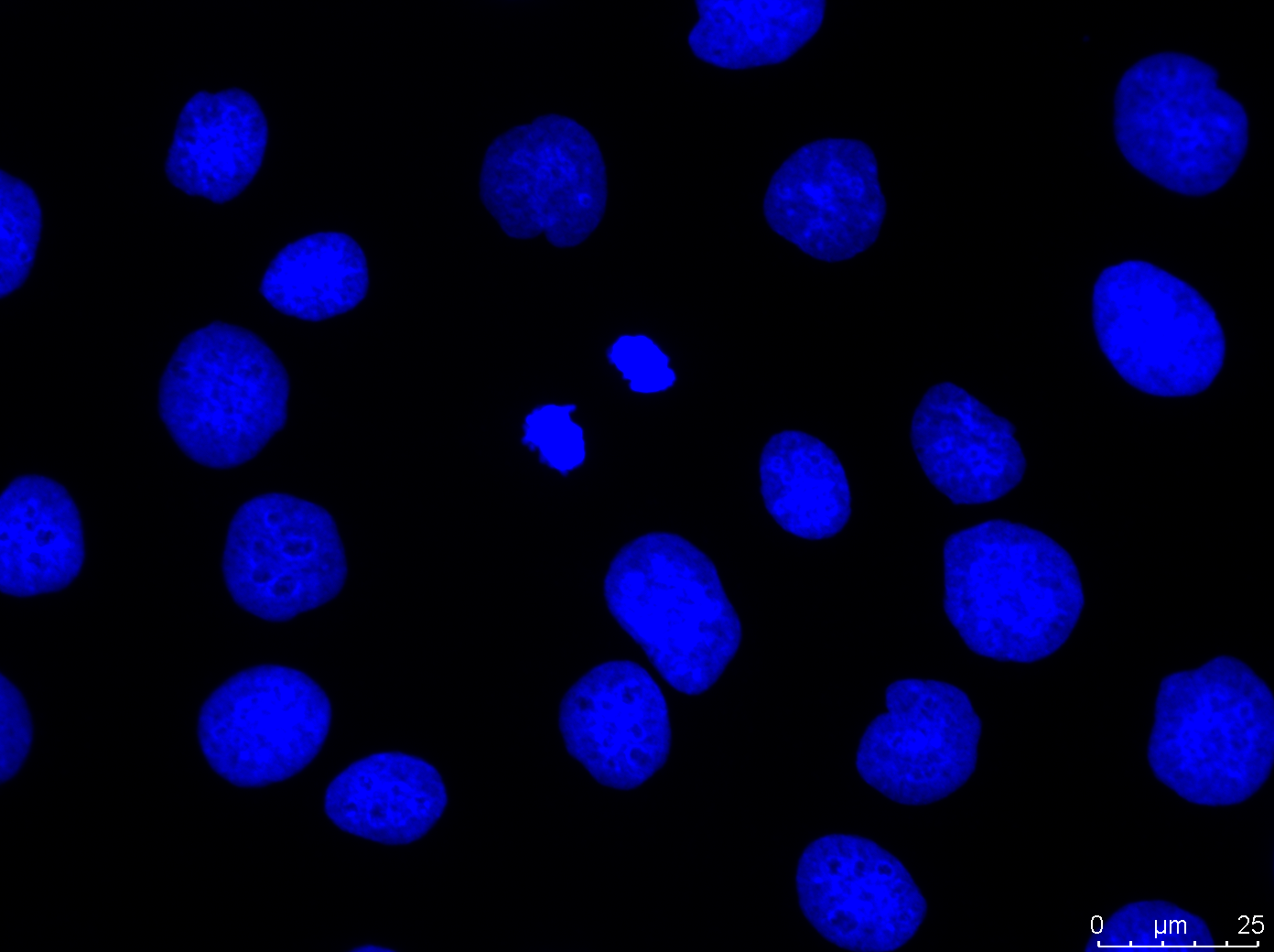

Supplement: Supplementary file 1 [file toxins-14-00785-s001.zip › to Fig 3_DAPI_Fluorescence_16HBE14o- Cells.tif]

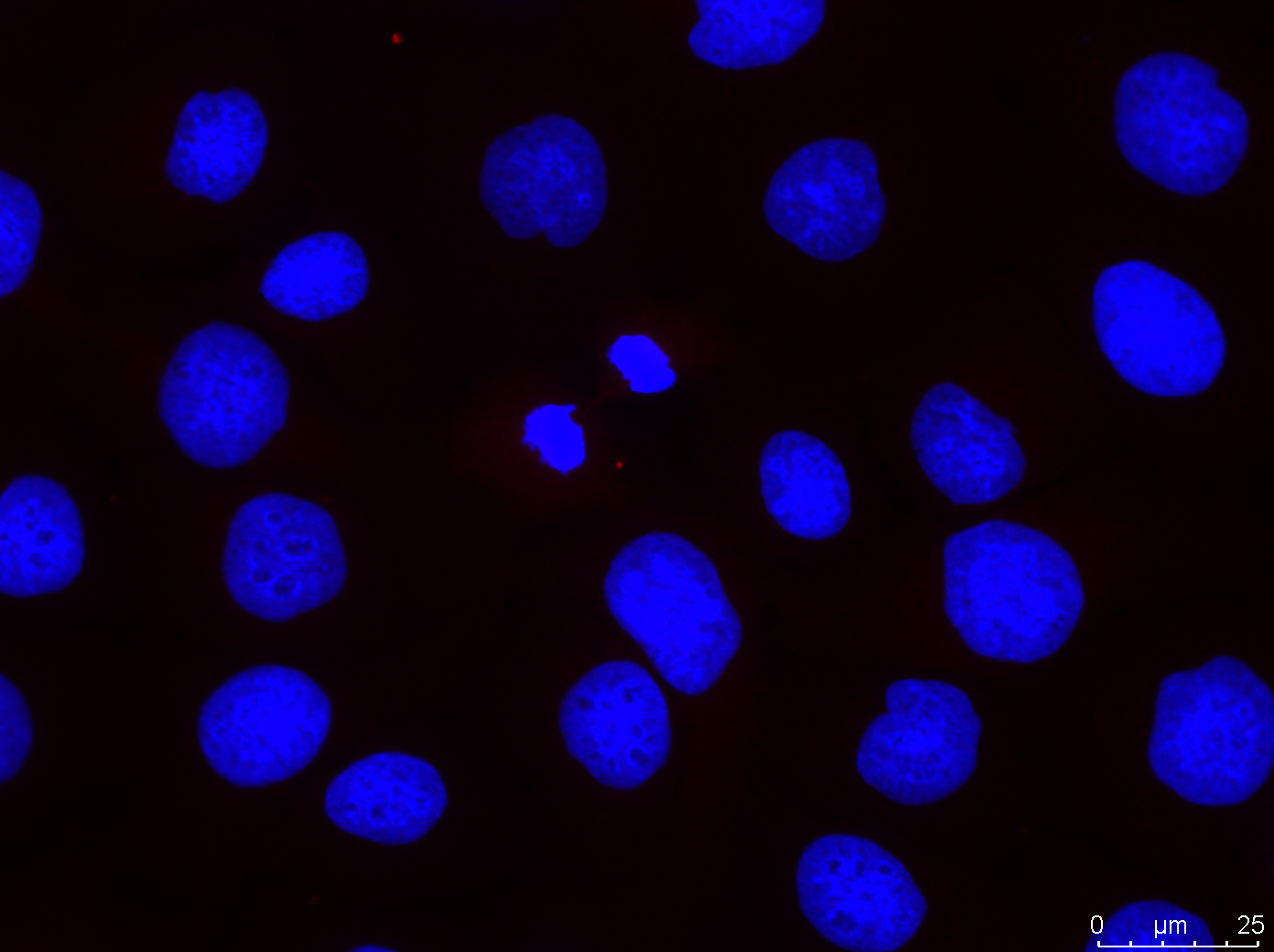

Supplement: Supplementary file 1 [file toxins-14-00785-s001.zip › to Fig 3_Overlay_CEP55_DAPI_Fluorescence_16HBE14o- Cells.tif]

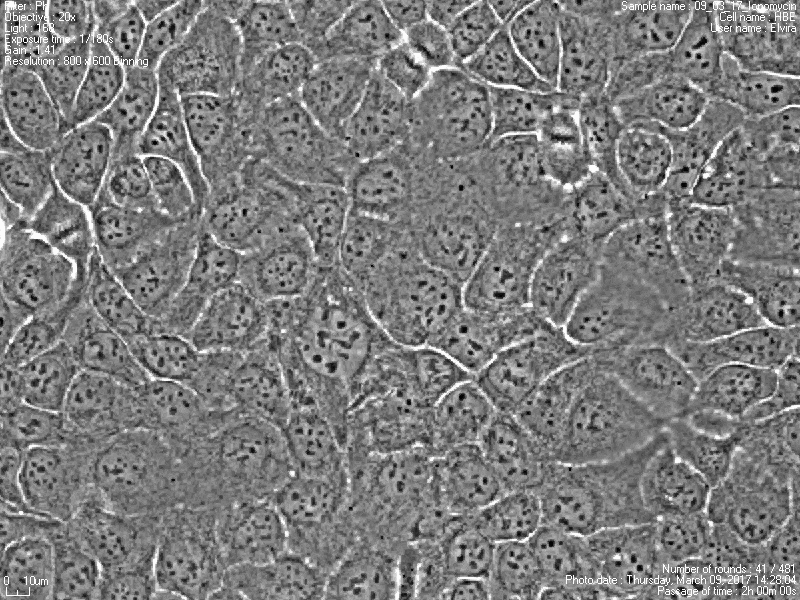

Supplement: Supplementary file 1 [file toxins-14-00785-s001.zip › to Suppl Fig 1_Time lapse still image_16HBE14o-_20x_Phase Contrast.tiff]
